# Semantically enabled and statistically supported biological hypothesis testing with tissue microarray databases

**DOI:** 10.1186/1471-2105-12-S1-S51

**Published:** 2011-02-15

**Authors:** Young Soo Song, Chan Hee Park, Hee-Joon Chung, Hyunjung Shin, Jihun Kim, Ju Han Kim

**Affiliations:** 1 Department of Industrial & Information Systems Engineering, Ajou University, Suwon 443-749, Korea; 2 Seoul National University Biomedical Informatics (SNUBI), Div. of Biomedical Informatics, Seoul National University College of Medicine, Seoul 110-799, Korea; 3 Systems Biomedical Informatics Research Center, Seoul National University, Seoul 110799, Korea

## Abstract

**Background:**

Although many biological databases are applying semantic web technologies, meaningful biological hypothesis testing cannot be easily achieved. Database-driven high throughput genomic hypothesis testing requires both of the capabilities of obtaining semantically relevant experimental data and of performing relevant statistical testing for the retrieved data. Tissue Microarray (TMA) data are semantically rich and contains many biologically important hypotheses waiting for high throughput conclusions.

**Methods:**

An application-specific ontology was developed for managing TMA and DNA microarray databases by semantic web technologies. Data were represented as Resource Description Framework (RDF) according to the framework of the ontology. Applications for hypothesis testing (Xperanto-RDF) for TMA data were designed and implemented by (1) formulating the syntactic and semantic structures of the hypotheses derived from TMA experiments, (2) formulating SPARQLs to reflect the semantic structures of the hypotheses, and (3) performing statistical test with the result sets returned by the SPARQLs.

**Results:**

When a user designs a hypothesis in Xperanto-RDF and submits it, the hypothesis can be tested against TMA experimental data stored in Xperanto-RDF. When we evaluated four previously validated hypotheses as an illustration, all the hypotheses were supported by Xperanto-RDF.

**Conclusions:**

We demonstrated the utility of high throughput biological hypothesis testing. We believe that preliminary investigation before performing highly controlled experiment can be benefited.

## Background

Biological databases are collections of scientific experiments, published literatures, and computational analyses organized under a specialized scheme. Biological databases became essential resources to biologists in their daily researches by providing information about biological facts and experimental results and procedures and also by providing management tools for the obtained data. Because these biological databases are designed for specific purposes, and independently managed, and metadata are not provided in many cases, they are neither semantically explicit nor interoperable. To overcome these problems, semantic web technologies such as Resource Description Framework (RDF), Web Ontology Language (OWL) and SPARQL (SPARQL Protocol and RDF Query Language) have been actively accepted in the field of life science for new database design [1-6]. Semantic web repositories are more advantageous than relational databases (RDBs) because metadata are more complete and standardized [7]. Representation of data as RDF makes biological entities semantically explicit and clear so that various tasks can be performed without extensive human interventions. These tasks includes integration of heterogeneous data, applying logic to infer new insights, and publication and sharing of biological findings and models [8]. Several current biological databases provide integrated data structure for knowledge management by applying semantic web technologies [1-4,6,9].

In spite of the benefits of semantic web technologies, these databases cannot directly answer to biologists for biologically meaningful questions or hypotheses. For machines to answer these questions, the process of inference based on either logical or statistical relationships of stored data is required. The inference by description logic is a part of semantic web technologies, but statistical inference was not implemented in the current semantic web technologies. These problems, therefore, cannot be solved within the framework of semantic web technologies alone and are rather dependent on the design of an application. However semantic web technologies are still beneficial in those applications.

To prove a biological hypothesis, 1) an experiment is designed to test the hypothesis, 2) the experimental data is gathered, and 3) the data is tested by statistical test(s). Because of the increasing amount of high throughput experimental data in biological databases, there is an increasing need of high throughput validation of biological hypotheses. To implement such an application, 1) a hypothesis given by a user should be semantically interpreted, 2) the relevant experimental data should be retrieved from the database, and then 3) the hypothesis should be statistically tested against the retrieved data.

Besides the fact that tissue microarray (TMA) is being widely used as a high throughput validation tool for the large number of data-driven hypotheses from other genomic technologies, TMA databases is a good candidate for the proof of concept of above mentioned applications. First, most biological hypotheses that can be derived from only TMA experiment are syntactically simple. The hypothesis derived from TMA experiments can be stated as, “In a biological condition A, an entity B is either positively or negatively correlated with an entity C.” Basically a TMA experiment is designed to test dependency between two entities and a hypothesis about the mechanisms of the interactions between two entities cannot be tested unless relevant additional information is provided. There are two important biological entities in TMA, biological samples and markers. In the TMA-validated hypothesis, “Reduced expression of Apaf-1 in colorectal cancer correlates with high-grade phenotype” [10], for example, ‘colorectal cancer’ is biological condition, and “reduced Apaf-1 expression” and “high-grade phenotype” are entities (B and C). Therefore a dependency-stating hypothesis in a TMA experiment can be considered as a triadic predicate with condition A, entity B, and entity C as parameters. If these parameters are provided, a query for relevant data retrieval can be generated through them. Second, only a few kinds of statistical tests are generally used to test hypothesis in TMA experiments. The main purpose of TMA experiment is to test the statistical relationships between biological entities (or markers) in a population of samples with identical biological condition. The results are determined by the size of the population conforming to the given hypothesis. If more samples show positive relationships with the hypothesis, the hypothesis is more likely to be true.

Fisher’s exact and χ^2^ test are the most frequently used statistical tests in TMA experiments to test dependency. They are frequently used because most of clinical or histological parameters (e.g., history of hypertension, tumour grade, etc) and the extent or the intensity of the marker expression in a tissue (e.g., 0, 1, 2, 3) have discrete or categorical values and the dependencies between these values are tested by them. For example, if we want to test the above mentioned hypothesis, “Reduced expression of Apaf-1 in colorectal cancer correlates with high-grade phenotype.” by Fisher’s exact test, we have to investigate each number of cores in slides for four exclusive conditions : a) negative Apaf-1 expression and high-grade phenotype, b) negative Apaf-1 expression and low-grade phenotype, c) weak to strong Apaf-1 expression and high-grade phenotype, and d) weak to strong Apaf-1 expression and low-grade phenotype. Then these four parameters are used for Fisher’s exact or χ^2^ test to test negative association between Apaf-1 expression and histological grade.

In spite of these benefits of TMA database, if it were not semantically explicit, applications for hypothesis testing could not be implemented. TMA data have complex and wide range of semantics, including information for clinical and histopathological features and large amount of metadata should be provided. Semantic web technologies support richer semantics than traditional RDB-based models. It, therefore, is more desirable that the databases for applications for hypothesis testing should be represented as RDF. In addition, SPARQL as a query language is more intuitively understandable to biologists [11]. Lastly, integration with the other databases, including other TMA and DNA microarray databases were considered in the present study and the databases using semantic web technologies are more advantageous in integration.

We have created and managed Xperanto-TMA, a web-based TMA database supporting TMA-OM (Tissue Microarray Object Model) [12] and TMA-TAB [13]. Xperanto-TMA uses RDB because technologies supporting object-oriented models were not mature enough to guarantee high performance [12]. Due to the well organized object model, however, clear and rich semantic relationships between entities are well supported. We have also developed and managed a web-based DNA microarray database called Xperanto [14].

When implementing Xperanto-TMA and Xperanto, we extensively investigated semantics of experimental data of TMA and DNA microarray. This experience gave us good resources in constructing semantically rich and explicit TMA and DNA microarray database represented as RDF. Because Xperanto-TMA and Xperanto were independently developed, it was difficult to integrate these two databases. The integration of gene expression microarray and TMA data is a powerful approach to molecular profiling of human cancer [15]. Semantic web gives us opportunity to integrate these two databases through the use of ontology [9]. After integration was achieved, we developed a use case where the usefulness of the integration was found.

In this study, we present how we designed semantic TMA database from the existing relational one and then how applications let researchers test hypotheses using semantic web infrastructures. Marker-level integration of TMA and DNA microarray databases will be also described.

## Methods

### RDF representation of TMA and DNA microarray data

#### Source databases

We represented Xperanto and Xperanto-TMA data as RDF triples. The relational schema were derived from TMA-OM by object-relational mapping [13]. The primary objective of Xperanto-TMA was to describe data and metadata from TMA experiments in a structured format with controlled vocabularies. Xperanto-TMA hosts more than 100 TMA experiments for various human cancers such as colon, gastric, and prostate cancers. For each experiment, detailed descriptions for clinical and histopathologic features including histology, grade, stage, and margin involvement are provided. Staining results are also provided for detailed data elements such as staining intensity and range.

Xperanto, which supports Microarray Gene Expression-Object Model (MAGE) [16], was developed to provide integrated data management and analysis for microarray data using user-friendly web-based interface [14]. Xperanto can store and analyze data and metadata from microRNA and ArrayCGH as well as DNA microarray experiment. It hosts more than 300 experiments for 52 array platforms.

In addition to these data, data about the mapping of DNA microarray probes to antibodies used in TMA experiment was provided using Genome Research Informatics Pipeline (GRIP, http://grip.snubi.org) to integrate these two databases.

#### Ontology design and data representation as RDF

**Figure 1:**
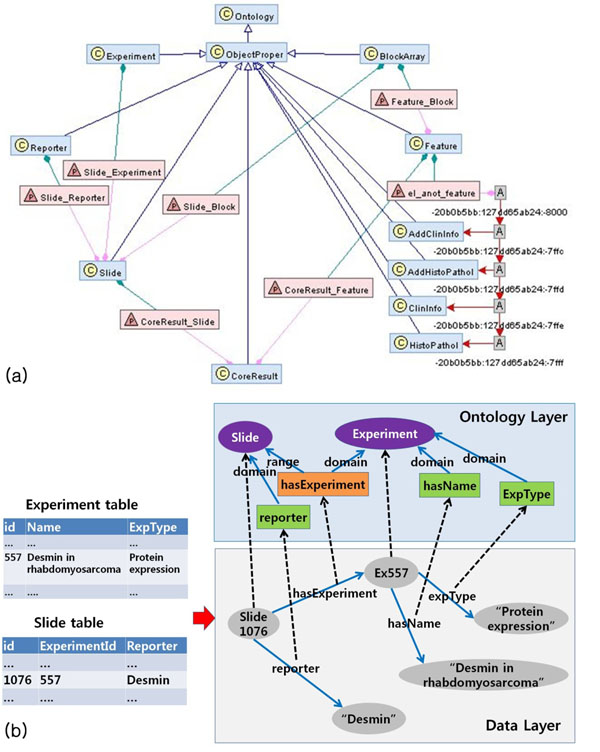
**Ontology and data representation as RDF** (a) A part of the ontology for main entities represented as RDF graphs. C (pale blue rectangles) represents classes, P (pink rectangles) properties, A anonymous nodes, empty arrows rdfs:subClassOf, pink arrows rdfs:domain, green arrows rdfs:range, and red arrows owl:unionOf. (b) Relationship between data tables in a source database (Xperanto-TMA) and data graphs represented as RDF graphs. Name spaces are omitted for simplicity. Violet ellipses represent classes, orange rectangles object properties, green rectangles data type properties, and dotted arrows class-instance relationships.

To provide RDF with a framework and facilitate the process of integration, an ontology specific for our applications was developed. In ontology design, our previous studies to implement Xperanto and Xperanto-TMA were referred because semantics in TMA and DNA microarray experiments were already extensively analyzed in the previous works [12,14]. Based on the previous works, the ontology was expressed as OWL. Part of the ontology is shown in Fig. 1(a) (Name spaces are omitted for simplicity).

Reuse of the existing ontologies such as NCI thesaurus or foundation model of anatomy (FMA) was not considered in this research because our main goal was to prove a hypothesis-testing system could be implemented with existing semantic web technologies and the goal could be achieved with our application-specific ontology.

According to the scheme of the ontology, we made a mapping rule for every data element (not shown) and data in Xperanto-TMA and Xperanto was represented as RDF. As in Fig. 1(b), for example, a row of Experiment table with three columns was transformed into two RDF triples. URIs for a subject were generated using the value of the primary key and the column names, “Name” and “ExpType” became predicates, “hasName” and “ExpType”. The values of the columns became objects of the triples. Each instance of URIs is an instance of an element (a class or a property) of the ontology. Note that a value of a foreign key (ExperimentId in Slide table) was transformed into an object of a triple, which was also a subject of another triple. In this way, all the data in the selected tables of source database could be represented as RDF triples.

#### System architecture

**Figure 2:**
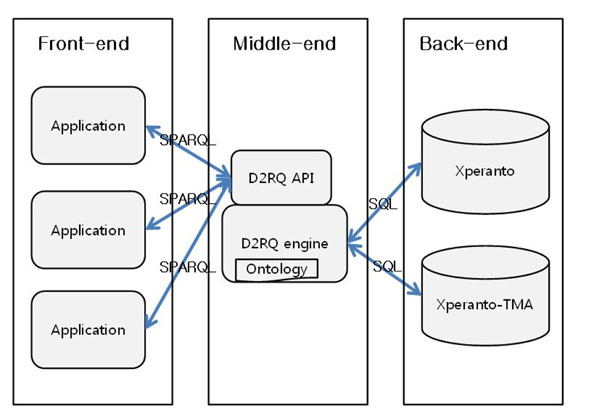
System architecture of Xperanto-RDF

In the actual implementation of RDF representation, we used a mediator system, D2RQ, instead of triple store for transformed RDF triples. When a mediator system is implemented, queries are directed against the mediator and then the mediator translates the queries into multiple SQLs for source RDBs. The use of mediator system have been shown to scale better and be easier to maintain [9]. D2RQ is a java API which mediates RDB and RDF-based applications according to an ontology and a mapping rule. The applications based on D2RQ can use SPARQL queries as if there were triple stores for the SPARQLs. The SPARQLs, then, is conveyed to D2RQ and D2RQ translates it into suitable SQL to the source databases. When query results are returned, D2RQ translate them into answers for the corresponding SPARQL and send it to applications (Fig. 2).

### Semantically-enabled hypothesis testing

#### Hypothesis model

We investigated the syntactic and semantic structures of four scientific hypotheses, which were validated by TMA experiments, from biomedical articles and pathologists. The hypotheses could be considered as triadic predicates with three parameters, “In a biological condition A, an entity B is either positively or negatively correlated with an entity C.” Factor A was defined as shared properties among the samples, factor B as an attribute and value set pair for distinct properties of the samples classifying the samples into two groups (the classifier), and factor C as an attribute and value set pair for dependent properties of the samples on the classifier.

In a hypothesis, “Reduced expression of Apaf-1 in colon cancer correlates with high-grade phenotype,” a shared property corresponded to colon cancer, a classifier to the reduced expression of Apaf-1 and dependent property to high histological grade. In this hypothesis, ‘reduced expression of Apaf-1’ needed to be explicitly stated through the process of the interpretation by pathologists and it was interpreted as the value of staining intensity of Apaf-1 in tumor cells having “0”. On the contrary, if “increased expression” had been used in the hypothesis, it could have been interpreted as the value having “1”, “2” or “3”, implying the complementary value set against “0”.

**Figure 3:**
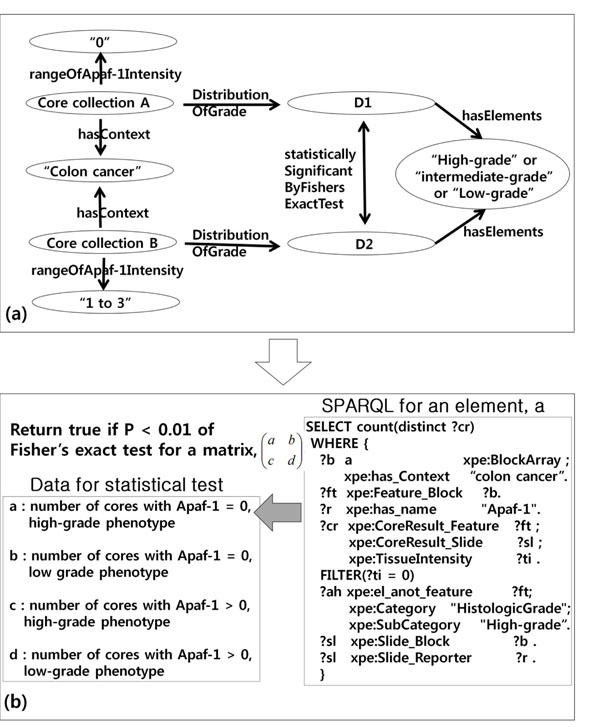
**Hypothesis model and procedures to test a hypothesis** (a) Model of a hypothesis, “Reduced expression of Apaf-1 in colorectal cancer correlates with high-grade phenotype”. (b) A statistical procedures for the hypothesis testing. Four SPARQLs are generated based on the hypothesis model (the other three SPARQLS not shown). The returned values of four SPARQLs are delivered as four elements for Fisher’s exact test.

Based on these findings, we made a model for the hypothesis to investigate how a given hypothesis can be analyzed with regards to TMA experimental data and statistical test (Fig. 3(a)). To get relevant data to the hypothesis, we needed data set where context was colon cancer and “Apaf-1 Intensity” and “HistologicGrade” were used as fields. Then we divided the selected data set into two groups, Core Collection A and Core Collection B, whose value for “Apaf-1 Intensity” were “0” and “1-3”, respectively. For each Core Collection A and B, we obtained the distribution of the values for “HistologicGrade”, which were designated as “D1” and “D2”, respectively. The element of distribution, D1 and D2 had a value, one among “High-grade”, “Intermediate-grade”, and “Low-grade”. The hypothesis would be supported if D1 and D2 were significantly different by Fisher’s exact test. The other three hypothesis models were constructed only by replacing the parameters of the above mentioned model (Table 1).

**Table 1:** Analysis of factors and their value sets of hypothesis and the results of statistical test (by Fisher’s exact test ) for the TMA data stored in Xperanto-RDF

Hypothesis	Shared property among samples	Classifier	Dependent property	Number of samples (cores) /slides/experiments found in Xperanto-RDF	P value
Reduced expression of Apaf-1 in colorectal carcinoma correlates with high-grade phenotype [10].	Colon cancer	Apaf-1 intensity (0 vs. 1-3)	Grade (High vs. Low-grade)	55/5/3	< 0.0001
In gastric cancer, Apaf-1 expression is associated with high grade malignancy.	Stomach caner	Apaf-1 intensity (0 vs. 1-3)	Grade (High vs. Low-grade)	52/4/3	< 0.0001
In colon cancer, the expression of Leptin is associated with negative lymph node metastasis [19].	Colon cancer	Leptin (positive vs. negative)	Nodal status (N0 vs N1-N3)	52/4/3	< 0.0001
In gastric cancer patients, HDAC2 expression was associated with negative lymph node metastasis [20].	Stomach caner	HDAC2 expression (positive vs. negative)	Nodal status (N0 vs N1-N3)	50/3/2	< 0.0001

The complementary value sets to the value sets of the classifier and the dependent property were parts of the hypothesis model, but they were not described in the hypothesis and should be reasoned. If a value set of either the classifier or the dependent property is provided, the complementary value set is generated as the complementary set to the value set of it among the set consisting of permissible value of the attribute of it. Taking an example from the above mentioned hypothesis, each of (“Low-grade”, “Intermediate-grade”) and ( “1”, “2”, “3”) was a complementary value set for the classifier and the dependent property, respectively because the permissible value lists for each factor were (“Low-grade”, “Intermediate-grade”, “High-grade”) and (“0”, “1”, “2”, “3”), and “High-grade” and “0” was described in the hypothesis. This process was implementable because we had already defined permissible value sets for every data element depending on the context when implementing Xperanto-TMA [12,13].

With the help of these analyses, we could implement the applications for hypothesis testing, which were operated in three steps. First the applications received inputs for the model construction. Second SPARQLs were generated according to the hypothesis model by a SPARQL generator. Third the applications called a statistical module and performed Fisher’s exact test using the result set of SPARQLs.

#### SPARQL generation

Four SPARQLs were generated from each hypothesis model by a SPARQL generator in the applications to obtain four elements for Fisher’s exact test. For example, the following is a SPARQL from the hypothesis model in Fig. 3(a) to get one element of 2-by-2 matrix for Fisher’s exact test, which is a number of cores with negative Apaf-1 expression and high-grade phenotype in colon cancer (Fig. 3(b)). The SPARQLs have four phrases in the WHERE clause and each phrase is independently generated using a transforming function in the SPARQL generator, Factor2SPARQL() having each factor and either hypothesis-describing or its complementary value set as parameters. The SPARQL is generated by concatenating these four phrases.

Generally, SPARQLs are generated as in the following.,

***SPARQL_i_*** := “SELECT count(distinct ?cr) WHERE {” + Σ***phrase_ij_*** + “?sl xpe:Slide Block ?b. ?sl xpe:Slide_Reporter ?r.}”

***Phrase_ij_*** := *Factor2SPARQL_j_*(***Factor_j_***, ***Valueset_ij_***)

***Valueset_ij_*** : if ***j*** = 1, the shared properties among the samples if ((***i*** = 1, 3) and ***j*** = 2) or ((***i*** = 1, 2) and ***j*** = 3), hypothesis-describing value set for ***Factor_j_***, otherwise its complementary value set

(***i*** = 1, 2, 3,4, ***j*** = 1, 2, 3).

#### Statistical test for Fisher’s exact test

After SPARQLs are processed, the applications send a list having four elements to the statistical module as four elements for Fisher’s exact test. The statistical module was made based on R (version 2.10.1., R Development Core Team, Vienna). After processing, the statistical module shows the p-value of the Fisher’s exact test to the user. Based on this p-value, the user can decide whether the database supports or rejects the given hypothesis.

### Use case of integrated TMA and DNA microarray database

We designed a use case using integrated DNA and TMA database, which was to find the distributions of the histologies among the cores of TMA where the intensities of the antibodies corresponding to the markers of interest in DNA microarray were high or low

## Results

### Xperanto-RDF

As described above, Xperanto-RDF was implemented based on existing TMA and DNA microarray databases (http://clara.snubi.org/Xperanto-RDF). Data from these two databases were represented as RDF triples by a mediator system such that users could have the benefits of semantic web technologies as well as those of RDB.

### Applications for hypothesis testing

**Figure 4:**
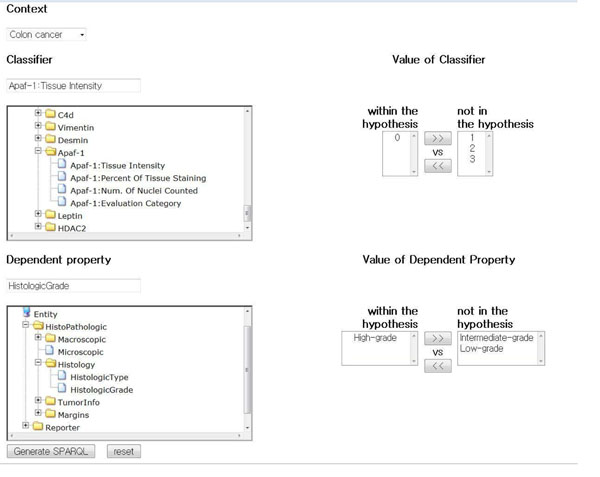
Web interface to generate SPARQLs by selecting factors and their value sets

A hypothesis model testing dependency can be constructed in the index page of Xperanto-RDF as shown in Fig. 4 (http://clara.snubi.org/Xperanto-RDF). In the context field, a user can choose the shared property among the samples. The attribute of the classifier and the dependent property can be selected by tracking along the tree structure of the entity. Once the classifier or the dependent property is selected, the permissible value set of the attribute is delivered to the select box to the right side of the tree frame. Using the buttons beside, the user can select hypothesis-describing and complementary value sets. Then the relevant SPARQL is generated by the SPARQL generator. On submitting the SPARQLs, the applications perform the query against the stored TMA data, the result sets are delivered to the statistical module, and finally the result of the statistical test including p-value is displayed.

As a result of hypothesis testing for the hypothesis in Fig. 3, we found the following results from the TMA database: 26 cores with Apaf-1 = 0 and high-grade phenotype, 4 cores with Apaf-1 ≥ 1 and high-grade phenotype, 6 core with Apaf-1 = 0 and low-grade phenotype, and 19 cores with Apaf-1 ≥ 1 and low-grade phenotype. These results support the given hypothesis because p value for Fisher’s exact text for these data was less than 0.0001.

Table 1 shows the testing results of all of four hypotheses. P value of the testing result determines whether the hypothesis is accepted or rejected according to Xperanto-RDF. With 0.01 of P value as a threshold for the rejection of null hypothesis, all of four hypotheses were accepted in these procedures. These results indicate that TMA data in Xperanto-RDF supports all of these hypotheses.

### Use case using integrated TMA and DNA microarray database

Xperanto-RDF achieved marker-mediated integration of TMA and DNA microarray data by mapping of DNA probes into antibodies in TMA experiment. By the use of mapping data, we could execute some of semantically integrated queries against data from TMA and DNA microarray experiments. A use case for this system was presented here.

*Query:* In an experiment for glioblastoma using Affymetrix HT Human Genome U133A array, the signal from the probe, “201983_s_at” were interesting to a researcher [17]. He wants to know the distribution of histologies of the samples where the corresponding antibody to the probe was highly expressed (intensity > 1) from our TMA database.

**Figure 5:**
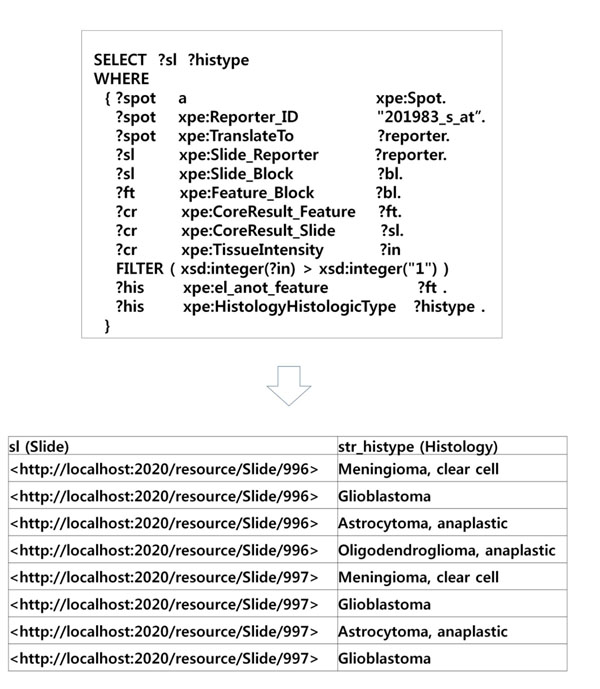
**An integrated query against TMA and DNA microarray database** This query returns a list of histological type of the samples which show strong intensity (>1) for the corresponding antibody to the mRNA probe, “201983_s_at”. The corresponding antibody is EGFR.

If TMA and DNA microarray database management systems had not been semantically integrated, it would have taken many steps to execute the queries and furthermore we needed external data source that showed what the corresponding antibody to the “201983_s_at” probe is. In Xperanto-RDF, the queries can be formulated as in Fig. 5. The query results show that the corresponding antibody is EGFR and the most predominant histological type among the samples where EGFR is highly expressed in TMA is glioblastoma according the data in Xperanto-RDF.

## Discussion

We presented how a semantic database for TMA and DNA microarray experiment can be developed from the existing RDB implementations using currently available semantic web technologies, how the application for hypothesis testing using TMA experimental data was designed and used, and how TMA database can be integrated with DNA microarray database using semantic web technologies.

We successfully implemented applications for hypothesis testing and their usefulness was validated by testing several experimentally validated biological hypotheses by TMA experiments. Although the applications seems useful to biologists, it is still challenging to spread the idea of the hypothesis testing in the biological database using experimental data to the whole community of the biologists. In traditional biomedical research, an experiment is designed to test a hypothesis [18] and it has been believed that only a highly controlled experiment could decide whether the hypothesis was true or false. The value of hypothesis testing in the biological database may be questionable from this point of view. Despite the presence of the weakness, the future of hypothesis testing using biological databases seems bright. More experiments are getting open to the public with detailed metadata about experiments and the experiments tend to be industrialized and standardized. As a result, it seems highly probable that a researcher can find most of the experiments they want from the biological databases in the near future. At present these applications can be coupled with traditional biomedical research in a cost effective way. A researcher can use these applications as a preliminary investigation before the highly controlled experiment is begun.

The applications for hypothesis testing can be regarded as a semantically-enhanced meta-analysis tool for TMA experiments in one aspect. One of requirements to perform meta-analysis is the process of data normalization. Our applications do not have the process of data normalization yet because there are no known methods of normalization in TMA experiments. The absence of normalization process do not appear to be greatly influential on the experimental results at present because TMA experiments are usually interpreted by human eyes, crude categorical values are used in the interpretation, and experimental procedures and interpretations are rather standardized.

We separated the process of hypothesis model construction, SPARQL execution, and statistical test when implementing the applications for hypothesis testing. This allows us more flexibility to easily manage and develop the whole systems. More statistical modules will be easily implemented to our applications if a hypothesis cannot be tested with current statistical modules. We will implement a statistical module for survival analysis soon.

Post et al. suggested integrative bioinformatics experimentation, defined as in silico data integration experiment using semantic web technologies, and made a use case to prove a hypothesis [7]. They tested a hypothesis stating that histone modification and gene expression regulation through transcription factor binding sites is correlated, by integrating histone modification data and transcription factor binding sites data derived from the public database using semantic web technologies [7]. Their integrative bioinformatics experimentation cycle consisted of hypothesis definition, experimental design, data integration, extension of data integration experiment, and data analysis and interpretation. Their study was focused on the applicability of semantic web technologies to data integration phase. Compared to this system, our applications process all the procedures to test the hypothesis after the hypothesis is inserted. To the user, the applications give impression as if the machine directly answered for the questions. We believe that we can implement the applications for hypothesis testing because we used TMA experiment data rather than genomic data and we targeted at the syntactically simple hypotheses composed of three factors. We believe that we can develop applications for testing for more complicated hypotheses based on this experience by treating complicated hypotheses as logical combinations of multiple simple hypotheses.

From the beginning our TMA database was developed with consideration on the integration with the other TMA or omics databases. Any TMA database based on TMA-OM can easily exchange the experimental data and the direct implementation of our application for hypothesis test is possible.

We also achieved marker-mediated integration of TMA and DNA microarray database. The integration enabled marker-mediated bidirectional data flow between TMA and DNA microarray experimental data. With these integrated databases, the results of DNA microarray experiments can be validated in TMA experiments.

Our future works are targeted in three directions. First, we are developing hypothesis miner extracting biological hypotheses directly from biomedical literature databases. The miner will feed hypotheses to TMA databases without the insertion procedures by the user. When realized, the integration of literature and experimental databases mediated by the hypotheses can be accomplished. Second, we will test if the same applications can be applied to DNA microarray databases using the same semantic web technologies. Although data-driven approach is predominant in interpreting the DNA microarray experiment results, we believe that traditional hypothesis-driven approach will continue to give us new insights to perceive the biological meaning of the DNA microarray experiment data. Third, we will improve descriptive power of our application-specific ontology and align it with existing ontologies. Our application specific ontology describes two array-based technologies (DNA and tissue microarray). Because DNA microarray is usually describable by MGED ontology, our ontology will become semantically more powerful by relating it with MGED ontology. Meanwhile, a part of our application-specific ontology was made based on TMA-OM and there is no other standard on ontology for TMA to the best of our knowledge. We, therefore, will expand our application-specific ontology so as to fully describe TMA experiment and align it with other neighbourhood ontologies.

## Conclusions

We proved semantic web technologies are beneficial in statistical inference as well as in logical inference based on description logic by implementing an application for hypothesis testing without disrupting previously implemented RDF-based applications. We proposed an application in which biologist’s real interests are reflected. The same scheme we used in developing Xperanto-RDF could be applied to the other omics technologies. However extensive study on the semantic and syntactic structure of the biologic hypothesis will more facilitate the development of applications for hypothesis testing in biological databases.

## Competing interests

All authors declared that there is no conflict of interest in this research.

## Authors' contributions

YSS and JHK designed and developed the study and wrote the manuscript. CHP and HJC implemented the system and wrote the manuscript. HJS and JK tested and coordinated the system and wrote the manuscript. All authors read and approved the final manuscript.
